# A Standardized Phlorotannin Supplement Attenuates Caffeine-Induced Sleep Disruption in Mice

**DOI:** 10.3390/nu11030556

**Published:** 2019-03-06

**Authors:** Sangoh Kwon, Minseok Yoon, Jaekwang Lee, Kwang-Deog Moon, Dohyeon Kim, Seon-Bong Kim, Suengmok Cho

**Affiliations:** 1 S&D Research and Development Institute, Cheongju 28156, Korea; so-kwon0004@hanmail.net (S.K.); concisenews@naver.com (D.K.); 2 Major in Food Biotechnology School of Food Science & Biotechnology, Kyungpook National University, Daegu 41566, Korea; kdmoon@knu.ac.kr; 3 Research Division of Food Functionality, Korea Food Research Institute, Wanju-gun 55365, Korea; msyoon@kfri.re.kr (M.Y.); jklee@kfri.re.kr (J.L.); 4 Department of Food Science and Technology, Pukyong National University, Busan 48513, Korea; owlkim@pknu.ac.kr

**Keywords:** phlorotannin, caffeine-induced arousal, sleep disruption, sleep-promoting, delta activity

## Abstract

In our previous studies, a standardized phlorotannin (brown seaweed polyphenol) supplement (PS) exhibited sleep-promoting effects via type A γ-aminobutyric acid-benzodiazepine receptors in mice. In addition, in human clinical trials, it decreased wake after sleep onset in adults with sleep disturbance. In this follow-up study, we investigated whether PS attenuates caffeine-induced sleep disruption in mice. The effects of PS were evaluated in a caffeine model by analyzing sleep architecture based on electroencephalogram and electromyogram findings, and were compared with the effects of a well-known sedative-hypnotic drug zolpidem (ZPD). As expected, oral administration of caffeine (25 mg/kg) significantly increased sleep latency and decreased the amount of non-rapid eye movement sleep (NREMS). In the caffeine + PS and caffeine + ZPD groups, PS (500 mg/kg) attenuated caffeine-induced sleep disruption, and its effects were comparable with those of ZPD (10 mg/kg). In particular, PS inhibited the arousal effects of caffeine without change in delta activity during NREMS, whereas ZPD produced a decrease in the delta activity. Considering global trends in coffee and energy drink consumption, our finding suggest that PS may be useful to relieve transitory insomnia symptoms caused by caffeine consumption, unlike the prescription drug ZPD.

## 1. Introduction

Phlorotannins, which exist within brown seaweeds, are oligomers and polymers of phloroglucinol (1,3,5-tri-hydroxybenzene) [1]. Marine polyphenol phlorotannins are structurally different from terrestrial polyphenols, which are based on gallic acids and flavones [2]. They exhibit high structural diversity similar to ginsenosides of ginseng [1,3,4]. To date, approximately 150 phlorotannin constituents have been identified, and individual phlorotannins show structural similarity to each other [5,6].

Phlorotannins are well-known as promising biologically active compounds. During the past two decades, the number of studies on the biological properties of phlorotannins has increased exponentially. They exhibit a variety of biological activities, including memory-enhancing [7], anti-oxidative [8], neuroprotective [9], anti-inflammatory [10], and anti-allergic [11] effects.

In our previous studies [12,13,14], we reported the sleep-promoting effects and action mechanism of phlorotannins. A standardized phlorotannin supplement (PS, 500 mg/kg, administered per os (p.o.)) significantly decreased sleep latency and increased non-rapid eye movement sleep (NREMS) in mice through positive allosteric modulation of the type A γ-aminobutyric acid (GABA_A_)-benzodiazepine receptors [13]. In addition, six major phlorotannin constituents (eckstolonol, triphlorethol A; eckol, fucodiphlorethol G; 6,6′-bieckol; and dieckol) were identified as hypnotic compounds. In a randomized, controlled, double-blind clinical and polysomnographic study, PS decreased wake after sleep onset in adults with self-reported sleep disturbance [14].

Caffeine, the major psychoactive constituent in coffee, disrupts physiological sleep and enhances arousal by inhibiting type 2A adenosine (A_2A_) receptors [15,16]. Accordingly, caffeine administration provides an effective model for the most common symptom of insomnia, difficulty in sleep initiation [16]. As a follow-up investigation to the aforementioned studies, in the present study, we evaluated the inhibitory effects of PS on caffeine-induced sleep disruption in mice through the analysis of electroencephalogram (EEG) and electromyogram (EMG) recordings.

## 2. Materials and Methods 

### 2.1. Materials

The PS (lot number: SD-GT-E-004) was obtained from S&D Co., Ltd. (Cheongju, South Korea). It was purified from the ethanol extract of the brown seaweed *Ecklonia cava* using a synthetic adsorbent resin (Diaion HP-20; Mitsubishi Chemical Industries Ltd., Tokyo, Japan), and was standardized to 67 mg/g dieckol. The total phlorotannin content of the PS, as assessed by the Folin-Ciocalteu method, was 90% (900 mg phloroglucinol equivalents/g). Zolpidem (ZPD), a GABA_A_-benzodiazepine agonist, was purchased from the Ministry of Food and Drug Safety of Korea (Cheongwon-gun, Chungcheongbuk-do, Korea), and was used as a reference hypnotic drug. Caffeine was purchased from Sigma-Aldrich, Inc. (St. Louis, MO, USA). All other chemicals and reagents were of the highest grade available.

### 2.2. Animals

All procedures involving animals were conducted in accordance with the animal care and use guidelines of the Korea Food Research Institutional Animal Care and Use Committee (permission number: KFRI-M-18005). C57BL/6N (male, weighing 27–30 g, 11 weeks old) mice were purchased from Koatech Animal, Inc. (Pyeongtaek, Korea). The animals were housed in an insulated, sound-proof recording room maintained at an ambient temperature of 23 ± 0.5 °C, with a constant relative humidity (55 ± 2%) and an automatically controlled 12 h light/dark cycle (lights off at 17:00). They had free access to food and water. All efforts were made to minimize animal suffering and to use the minimum number of animals required for the acquisition of reliable scientific data.

### 2.3. Pharmacological Treatments

All drugs were dissolved in sterile saline containing 5% tween 80 immediately before use, and administered orally (p.o.) to the mice (n = 7–8 per group) using a sonde needle at 17:00 on the experimental day. For baseline data, mice were administered the vehicle (saline containing 5% tween 80) at 17:00 (p.o.).

### 2.4. Analysis of Sleep Architecture

**Figure 1:**
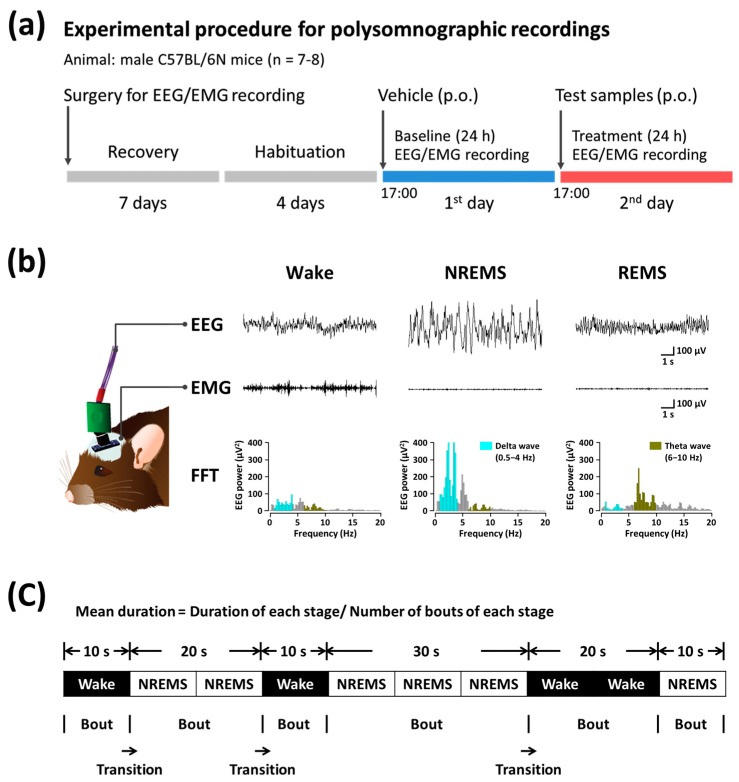
Experimental procedures and timelines for the polysomnographic recordings (**a**) and typical EEG and EMG waveforms, and FFT spectrum in mice (**b**); (**c**) Definition of sleep-wake episodes. EEG, electroencephalogram; EMG, electromyogram; FFT, fast Fourier transform; REMS, rapid eye movement sleep; NREMS, non-REMS; Wake, wakefulness.

Sleep analysis was performed using the method of Um et al. [17]. The experimental procedure and timeline for sleep analysis is shown in Figure 1a. Under pentobarbital anesthesia (50 mg/kg, administered intraperitoneally), the mice were chronically implanted with a head mount (#8201; Pinnacle Technology, Inc., Lawrence, KS, USA) installed with EEG and EMG electrodes for polysomnographic recordings. The front edge of the head mount was placed 3.0 mm anterior to the bregma, and four electrode screws for EEG recording were positioned in holes perforated into the skull. Two EMG wire electrodes were inserted into the nuchal muscles. The head mount was fixed to the skull with dental cement. After surgery, the mice were allowed to recover in individual cages for 1 week, and allowed to acclimate to the recording conditions for 3–4 days before the experiment. The EEG and EMG recordings were performed by means of a slip ring designed such that the movement of the mice was not restricted. The EEG and EMG signals were recorded using the PAL-8200 data acquisition system (Pinnacle Technology, Inc.). The EEG and EMG signals were amplified (100×), filtered (low-pass filter: 25 Hz for EEG and 100 Hz for EMG), and stored at a sampling rate of 200 Hz. Sleep-wake states were monitored for a period of 48 h, which included baseline data acquisition and experimentation. Baseline recordings were obtained for each animal over 24 h, beginning at 17:00. These baseline recordings served as control for the same animal. Mice were considered asleep when no EMG signal was detectable. The vigilance states were automatically classified by a 10-s epoch as wakefulness (Wake), rapid eye movement sleep (REMS), or non-REM sleep (NREMS) using SleepSign version 3.0 (Kissei Comtec, Nagano, Japan). As a final step, the defined sleep-wake stages were examined visually and corrected if necessary. The sleep latency was defined as the time from drug administration to the appearance of the first NREMS episode lasting for at least 120 s. In each instance, the delta power during NREMS in the range of 0.5–4 Hz was first summated and then normalized as a percentage of the corresponding mean delta power during NREMS. Representative EEG/EMG waveforms and fast Fourier transform spectra of delta and theta waves are shown in Figure 1b. Bouts of NREMS, REMS, and Wake were defined as periods of one or more consecutive epochs (each epoch: 10 s) (Figure 1c).

### 2.5. Data Analysis

All data are expressed as the mean ± standard error of the mean. Statistical analysis was performed using the Prism 5.0 software (GraphPad Software, Inc., San Diego, CA, USA). Comparisons between two groups were performed using the unpaired Student’s *t*-test. The significance level was set at *p* < 0.05.

## 3. Results

### 3.1. Effects of PS and ZPD on Sleep Latency and NREMS in Caffeine-Induced Sleep Disruption

**Figure 2:**
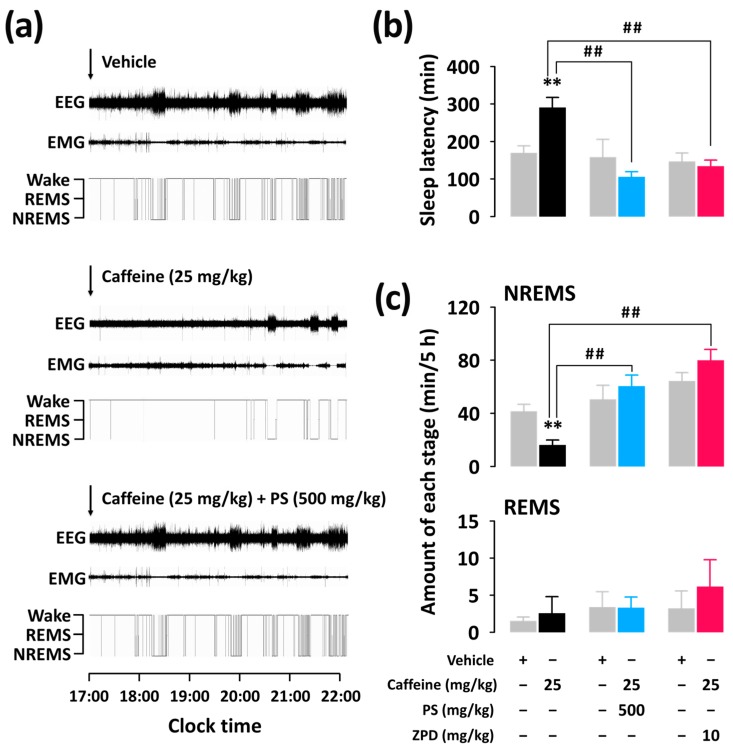
Sleep-wake profiles in C57BL/6N mice after oral administration of caffeine alone and co-administration of PS or ZPD with caffeine. (**a**) Typical EEG and EMG recordings and the corresponding hypnograms in a mouse treated with vehicle, caffeine, or caffeine + PS. (**b**) Effects of caffeine, caffeine + PS, and caffeine + ZPD on sleep latency. (**c**) Amount of NREMS and REMS during the 5-h period after administration of vehicle, caffeine, caffeine + PS, and caffeine + ZPD. Grey bars indicate the baseline day (vehicle). Each value represents the mean ± standard error of the mean of 7–8 mice per group. ** *p* < 0.01, significantly different from the vehicle control and ## *p* < 0.01, significant difference between two groups (unpaired Student’s *t*-test). EEG, electroencephalogram; EMG, electromyogram; NREMS, non-rapid eye movement sleep; PS, a standardized phlorotannin supplement; REMS, rapid eye movement sleep; Wake, wakefulness; ZPD, zolpidem.

Figure 2a presents typical examples of polysomnographic recordings and corresponding hypnograms from a single mouse during the first 5 h following vehicle, caffeine, and caffeine + PS or ZPD administration. Dosages of caffeine (25 mg/kg), PS (500 mg/kg), and ZPD (10 mg/kg) were selected based on preliminary experiments. The value of the sleep latency was 290.2 ± 26.9 min in mice treated with caffeine (25 mg/kg) (Figure 2b). This value was significantly (*p* < 0.01) longer than that of sleep latency after vehicle treatment, which was observed to be 169.3 ± 18.7 min. However, the sleep latency for oral administration of caffeine (25 mg/kg) + PS (500 mg/kg) and caffeine (25 mg/kg) + ZPD (10 mg/kg) was not significantly different compared with that for each vehicle. We calculated the amounts of NREMS and REMS during the first 5 h after the administrations of caffeine, caffeine + PS, and caffeine + ZPD (Figure 2c). As expected, the caffeine (25 mg/kg) decreased the amount of NREMS by 2.6-fold (*p* < 0.01) compared with the vehicle. Co-administration of PS or ZPD with caffeine producing significantly (*p* < 0.01) longer NREMS than that of the group administered caffeine alone. There was no significant difference in REMS for all groups. 

**Figure 3:**
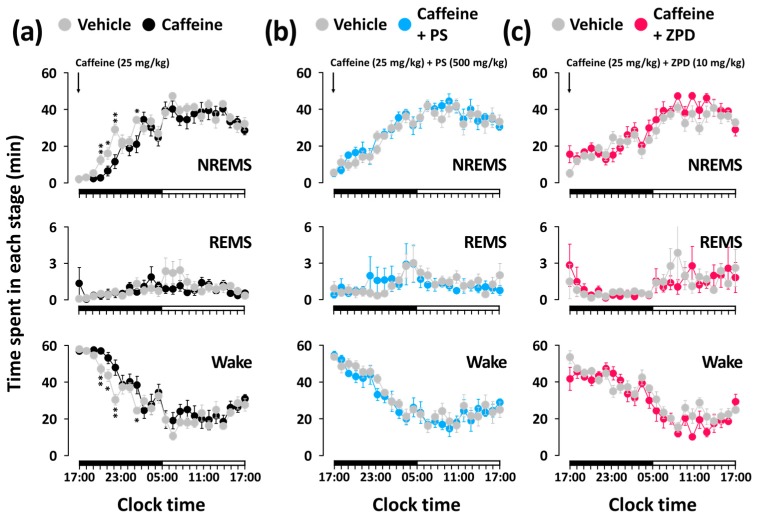
Temporal changes in NREMS, REMS, and Wake in C57BL/6N mice over 24 h after oral administration (per os) of caffeine alone (**a**) and co-administration of PS (**b**) or ZPD (**c**) with caffeine. Grey circles indicate the baseline day (vehicle administration). Each circle represents the hourly mean ± standard error of the mean amount of each stage (n = 7–8 per group). * *p* < 0.05 and ** *p* < 0.01, significantly different from the vehicle control (unpaired Student’s *t*-test). The horizontal filled and open bars on the X-axis (clock time) indicate the 12 h dark and light periods, respectively. NREMS, non-rapid eye movement sleep; PS, a standardized phlorotannin supplement; REMS, rapid eye movement sleep; Wake, wakefulness; ZPD, zolpidem.

Figure 3 shows the time course of NREMS, REMS, and Wake for 24 h after the administration of caffeine, caffeine + PS, and caffeine + ZPD. Caffeine significantly decreased the amount of NREMS during the fourth, fifth, sixth, and ninth hours after administration by 4.59-, 2.49-, 2.52-, and 1.63-fold compared with the vehicle, respectively (Figure 3a). This reduction of NREMS was accompanied by a significant increase in Wake during the same time. Unlike the caffeine-only group, both caffeine + PS and caffeine + ZPD groups did not exhibit a change in sleep architecture for 24 h (Figure 3b,c). 

### 3.2. Effects of PS and ZPD on Characteristics of Sleep–Wake Episodes and EEG Power Density in Caffeine-Induced Sleep Disruption

**Figure 4:**
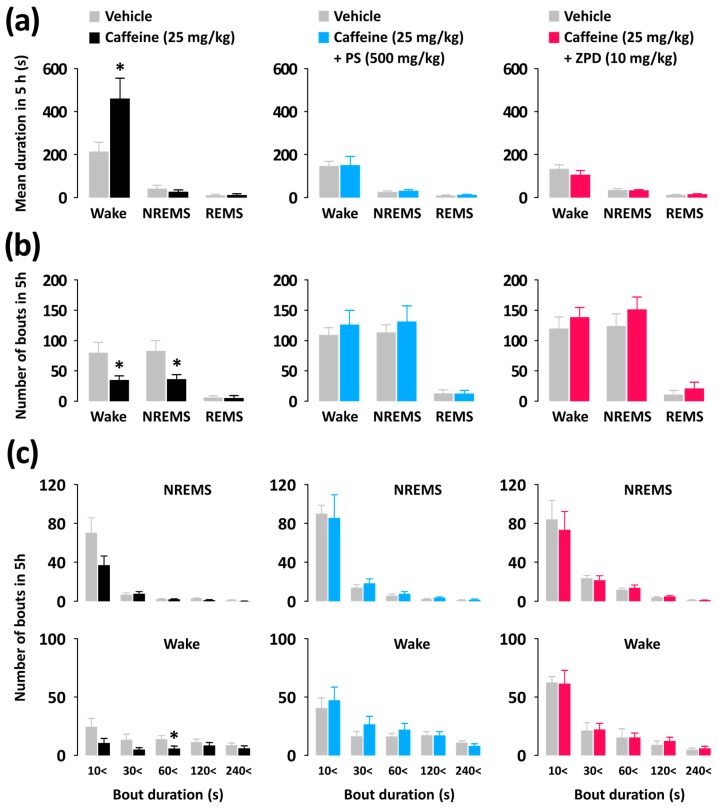
Characteristics of sleep-wake episodes in C57BL/6N mice during the 5 h period after administration of caffeine alone and co-administration of PS or ZPD with caffeine. (**a**) Changes in the mean duration of Wake, NREMS, and REMS bouts. (**b**) Changes in the total number of Wake, NREMS, and REMS bouts. (**c**) Changes in the numbers of NREMS and Wake bouts for different ranges of duration. Grey bars indicate the baseline day (vehicle). Each value represents the mean ± standard error of the mean of each group (n = 7–8). * *p* < 0.05, significantly different from the vehicle control (unpaired Student’s *t*-test). NREMS, non-rapid eye movement sleep; PS, a standardized phlorotannin supplement; REMS, rapid eye movement sleep; Wake, wakefulness; ZPD, zolpidem.

**Figure 5:**
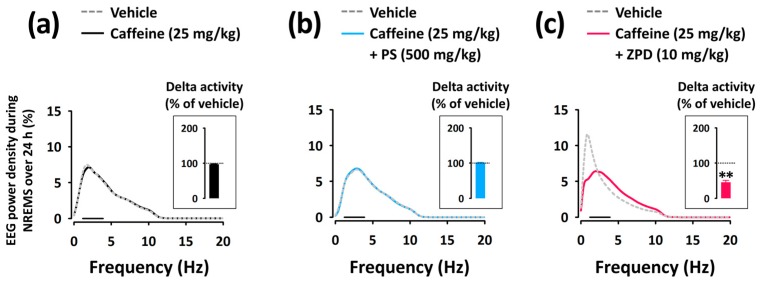
EEG power density curves during NREMS caused by caffeine alone (**a**) and co-administration of PS (**b**) or ZPD (**c**) with caffeine. Delta activity, an index of sleep intensity, is shown in the inset histogram. The solid bar (▬) represents the range of the delta wave (0.5‒4 Hz). ** *p* < 0.01, significantly different from the vehicle control (unpaired Student’s *t*-test). EEG, electroencephalogram; NREMS, non-rapid eye movement sleep; PS, a standardized phlorotannin supplement; ZPD, zolpidem.

To better understand the nature of the arousal-inhibiting effects of PS and ZPD, we additionally analyzed the mean duration and total number of NREMS, REMS, and Wake episodes, as well as EEG power density (Figure 4 and Figure 5). Caffeine (25 mg/kg) significantly increased the mean duration of Wake by 146.4% (*p* < 0.05), without affecting that of NREMS or REMS (Figure 4a). Moreover, caffeine (25 mg/kg) decreased the number of Wake and NREMS bouts by 2.3, respectively (Figure 4b), and decreased the number of bouts of Wake that ranged from 60 to 120 s (Figure 4c). On the other hand, co-administration of PS (500 mg/kg) or ZPD (10 mg/kg) with caffeine (25 mg/kg) did not show significant difference in the mean duration and total number of NREMS, REMS, and Wake episodes compared with each vehicle.

To evaluate the sleep quality, we analyzed the EEG power density in mice during NREMS, and measured the delta activity. As shown in Figure 5a,b, caffeine and caffeine + PS did not affect the EEG power density (0–20 Hz), including delta activity (frequency range of 0.5–2.5 Hz), in NREMS compared with each vehicle. Co-administration of ZPD with caffeine, however, significantly (*p* < 0.01) decreased the delta activity (Figure 5c).

## 4. Discussion

In the present study, we investigated whether PS shows sleep-promoting effects in a caffeine-induced sleep disruption mouse model. The effects of PS on caffeine-induced arousal were compared with those of the well-known sedative-hypnotic ZPD. In our previous study [13], PS (> 250 mg/kg, p.o.) significantly increased NREMS during the first 2 h after administration and decreased sleep latency in a normal animal model (C57BL/6N mice) without caffeine treatment. In addition, it was demonstrated that PS promotes sleep by modulating the GABA_A_-benzodiazepine receptors.

Caffeine is one of the most widely consumed food substances in the world, and is mostly consumed as coffee and caffeinated beverages, including energy drinks [18]. The effects of caffeine on sleep, both on humans and rodents, are well-demonstrated [16]. Generally, caffeine administration produces increased sleep latency and reduced total sleep time and efficiency [16]. These arousal effects of caffeine may exert a beneficial effect against sleep deprivation or daytime sleepiness; however, it exerts sleep-disruptive effects on subsequent nights [19]. The disruptive effects of caffeine on sleep may be broadly classified as insomnia symptoms and abnormalities of sleep disruption [19]. Therefore, the caffeine-induced arousal model appears well-suited to reproduce the underlying feature of insomnia [16]. In addition, the caffeine-induced sleep disruption model possesses advantages that make it an excellent insomnia model: it is simple and cost-effective, is widely used and safe, and can be used both in animals and humans [16].

In the present study, as expected, caffeine (25 mg/kg) significantly increased sleep latency (Figure 2b) and decreased NREMS during the first 5 h after administration (Figure 2c). These sleep-disruptive effects of caffeine were reported by Huang et al. [15]. It was confirmed that caffeine (15 mg/kg) increased Wake in mice during the first 3 h after administration. When PS (500 mg/kg) or ZPD (10 mg/kg) was co-administered with caffeine (25 mg/kg), significant arousal effects of caffeine were not observed (Figure 2b,c). These results imply that PS and ZPD, which exert sedative-hypnotic effects, attenuate caffeine-induced arousal in mice. According to the report by Paterson et al. [20], oral co-administration of ZPD (10 mg/kg) with caffeine significantly attenuates the caffeine-induced increase in sleep latency in both rats and humans. In the study by Paterson et al. [20], caffeine and hypnotic drugs were administered to rats in the light phase (14:00) corresponding to the resting phase, whereas drug administration in our study was performed in the dark phase (17:00) corresponding to the active phase. Normal sleep in rodents is fragmented, and sleep and wake appear during both light and dark phases [16]. Therefore, for evaluating hypnotic and arousal effects in rodents, test drugs can be administered during both light and dark periods. For assessing attenuating effects of hypnotic agents on caffeine-induced sleep disruption, administration in the dark phase, when rodents are active and under low sleep pressure may be appropriate [21,22]. It is generally accepted that the arousal effects of caffeine are sensitive to different types of sedative-hypnotic agents, such as temazepam, zolpidem, or trazodone, in rodents and humans [16]. These sedative-hypnotic agents attenuate or reverse caffeine-induced arousal. Both PS and ZPD induce sleep by agonizing the GABA_A_-benzodiazepine receptors, and caffeine promotes Wake by antagonizing the adenosine A_2A_ receptors in the central nervous system. Therefore, the inhibitory effects of PS and ZPD on caffeine-induced arousal imply that they may counteract the effect of caffeine on sleep disturbance, but not by competing with the binding of caffeine to the adenosine receptors. 

Interestingly, in the present study, both PS and ZPD reversed caffeine-induced sleep disruption; however, there was one difference between the effect of these compounds: PS did not produce changes in EEG power density during NREMS compared with the vehicle (Figure 5b), whereas ZPD significantly decreased the delta activity (the range of 0.5–4.0 Hz in EEG power density) (Figure 5c). In Figure 5, three EEG power density curves of vehicle groups show different shapes. This difference in the EEG power density curves originates from differences in EEG waves of individual mice. Similar results have also been reported by other rodent EEG studies [23,24]. Delta activity is an indicator of the quality or intensity of NREMS [25,26]. In humans and rodents, ZPD, the GABA_A_-benzodiazepine receptor agonist, increases the sleep quantity in NREMS but reduces the delta activity [25,27]. Yang et al. [28] reported that ZPD (10 mg/kg) significantly decreases delta activity in normal C57BL/6N mice without caffeine treatment. From the results of the present study, we found that ZPD also produces a typical decrease in delta activity in a caffeine-induced sleep disruption model.

## 5. Conclusions

Despite a global increase in caffeine consumption, few studies have been conducted on foods and natural products attenuating caffeine-induced sleep disruption. In the present study, we found that marine polyphenol PS attenuated caffeine-induced arousal similarly to the sedative-hypnotic drug ZPD. Unlike the prescription drug ZPD, PS may be useful to relieve the transitory insomnia symptoms caused by caffeine consumption. In particular, PS showed an advantage over ZPD in that it preserves the intensity of NREMS. Considering the global trends in coffee and energy drink consumption, the attenuating effect of PS on caffeine-mediated sleep disruption adds to its value as a natural sleep aid.

## References

[1] Shibata T., Kawaguchi S., Hama Y., Inagaki M., Yamaguchi K., Nakamura T. (2004). Local and chemical distribution of phlorotannins in brown algae. J. Appl. Phycol..

[2] Shibata T., Fujimoto K., Nagayama K., Yamaguchi K., Nakamura T. (2002). Inhibitory activity of brown algal phlorotannins against hyaluronidase. Int. J. Food Sci. Technol..

[3] Ramarajan L., Somasundaram S.T., Subramanian S., Pandian V. (2012). Nephroprotective effects of *Colpomenia sinuosa* (Derbes & Solier) against carbon tetrachloride induced kidney injury in Wistar rats. Asian Pac. J. Trop. Dis..

[4] Xiang Y.Z., Shang H.C., Gao X.M., Zhang B.L. (2008). A comparison of the ancient use of ginseng in traditional Chinese medicine with modern pharmacological experiments and clinical trials. Phytother. Res..

[5] Martínez J.H.I., Castañeda H.G.T. (2013). Preparation and chromatographic analysis of phlorotannins. J. Chromatogr. Sci..

[6] Koivikko R., Loponen J., Pihlaja K., Jormalainen V. (2007). High-performance liquid chromatographic analysis of phlorotannins from the brown alga *Fucus Vesiculosus*. Phytochem. Anal..

[7] Myung C.S., Shin H.C., Bao H.Y., Yeo S.J., Lee B.H., Kang J.S. (2005). Improvement of memory by dieckol and phlorofucofuroeckol in ethanol-treated mice: Possible involvement of the inhibition of acetylcholinesterase. Arch. Pharm. Res..

[8] Zou Y., Qian Z.J., Li Y., Kim M.M., Lee S.H., Kim S.K. (2008). Antioxidant effects of phlorotannins isolated from *Ishige okamurae* in free radical mediated oxidative systems. J. Agric. Food Chem..

[9] Yoon N.Y., Chung H.Y., Kim H.R., Choi J.S. (2008). Acetyl-and butyrylcholinesterase inhibitory activities of sterols and phlorotannins from *Ecklonia stolonifera*. Fish. Sci..

[10] Kim A.R., Shin T.S., Lee M.S., Park J.Y., Park K.E., Yoon N.Y., Kim J.S., Choi J.S., Jang B.C., Byun D.S. (2009). Isolation and identification of phlorotannins from *Ecklonia stolonifera* with antioxidant and anti-inflammatory properties. J. Agric. Food Chem..

[11] Shim S.Y., Quang-To L., Lee S.H., Kim S.K. (2009). *Ecklonia cava* extract suppresses the high-affinity IgE receptor, FcεRI expression. Food Chem. Toxicol..

[12] Cho S., Yang H., Jeon Y.J., Lee C.J., Jin Y.H., Back N.I., Kim D.S., Kang S.M., Yoon M., Yong H. (2012). Phlorotannins of the edible brown seaweed *Ecklonia cava* Kjellman induce sleep via positive allosteric modulation of gamma-aminobutyric acid type A-benzodiazepine receptor: A novel neurological activity of seaweed polyphenols. Food Chem..

[13] Cho S., Yoon M., Pae A.N., Jin Y.H., Cho N.C., Takata Y., Urade Y., Kim S., Kim J.S., Yang H. (2014). Marine polyphenol phlorotannins promote non-rapid eye movement sleep in mice via the benzodiazepine site of the GABA_A_ receptor. Psychopharmacology.

[14] Um M.Y., Kim J.Y., Han J.K., Kim J., Yang H., Yoon M., Kim J., Kang S.W., Cho S. (2018). Phlorotannin supplement decreases wake after sleep onset in adults with self-reported sleep disturbance: A randomized, controlled, double-blind clinical and polysomnographic study. Phytother. Res..

[15] Huang Z.L., Qu W.M., Eguchi N., Chen J.F., Schwarzschild M.A., Fredholm B.B., Urade Y., Hayaishi O. (2005). Adenosine A_2A_, but not A_1_, receptors mediate the arousal effect of caffeine. Nat. Neurosci..

[16] Revel F.G., Gottowik J., Gatti S., Wettstein J.G., Moreau J.L. (2009). Rodent models of insomnia: A review of experimental procedures that induce sleep disturbances. Neurosci. Biobehav. Rev..

[17] Um M.Y., Kim S., Jin Y.H., Yoon M., Yang H., Lee J., Jung J., Urade Y., Huang Z.L., Kwon S. (2017). A novel neurological function of rice bran: A standardized rice bran supplement promotes non-rapid eye movement sleep in mice through histamine H_1_ receptors. Mol. Nutr. Food Res..

[18] McLellan T.M., Caldwell J.A., Lieberman H.R. (2016). A review of caffeine’s effects on cognitive, physical and occupational performance. Neurosci. Biobehav. Rev..

[19] Chaudhary N.S., Grandner M.A., Jackson N.J., Chakravorty S. (2016). Caffeine consumption, insomnia, and sleep duration: Results from a nationally representative sample. Nutrition.

[20] Paterson L.M., Wilson S.J., Nutt D.J., Hutson P.H., Ivarsson M. (2007). A translational, caffeine-induced model of onset insomnia in rats and healthy volunteers. Psychopharmacology.

[21] Depoortere H., Decobert M., Granger P., Riou-Merle F. (1986). Hypnotics: Clinical value of pharmaco-EEG methods. Neuropsychobiology.

[22] Saitou K., Kaneko Y., Sugimoto Y., Chen Z., Kamei C. (1999). Slow wave sleep-inducing effects of first generation H_1_-antagonists. Biol. Pharm. Bull..

[23] Qiu M.H., Qu W.M., Xu X.H., Yan M.M., Urade Y., Huang Z.L. (2009). D_1_/D_2_ receptor-targeting L-stepholidine, an active ingredient of the Chinese herb *Stephonia*, induces non-rapid eye movement sleep in mice. Pharmacol. Biochem. Behav..

[24] Huang Z.L., Mochizuki T., Qu W.M., Hong Z.Y., Watanabe T., Urade Y., Hayaishi O. (2006). Altered sleep-wake characteristics and lack of arousal response to H_3_ receptor antagonist in histamine H_1_ receptor knockout mice. Proc. Natl. Acad. Sci. USA.

[25] Tobler I., Kopp C., Deboer T., Rudolph U. (2001). Diazepam-induced changes in sleep: Role of the α_1_ GABA_A_ receptor subtype. Proc. Natl. Acad. Sci. USA.

[26] Chen C.R., Zhou X.Z., Luo Y.J., Huang Z.L., Urade Y., Qu W.M. (2012). Magnolol, a major bioactive constituent of the bark of *Magnolia officinalis*, induces sleep via the benzodiazepine site of GABA_A_ receptor in mice. Neuropharmacology.

[27] Feinberg I., Maloney T., Campbell I.G. (2000). Effects of hypnotics on the sleep EEG of healthy young adults: New data and psychopharmacologic implications. J. Psychiatr. Res..

[28] Yang H., Yoon M., Um M.Y., Lee J., Jung J., Lee C., Kim Y.T., Kwon S., Kim B., Cho S. (2017). Sleep-promoting effects and possible mechanisms of action associated with a standardized rice bran supplement. Nutrients.

